# Case Finding Among and Comprehensive Management of Household Contacts of Persons with Pulmonary Tuberculosis: a Pilot Project — Uganda, 2023–2024

**DOI:** 10.15585/mmwr.mm7409a1

**Published:** 2025-03-20

**Authors:** Denis Mudoola, Pruthu Thekkur, Joseph Nsonga, Ritah Mande, Selma Dar Berger, Stavia Turyahabwe, Simon Muchuro, Proscovia Namuwenge, Moorine Sekadde, Deus Lukoye, Henry Luzze, John Paul Dongo, Anand Date, Riitta A. Dlodlo, Odile Ferroussier-Davis, Macarthur Charles

**Affiliations:** ^1^International Union Against Tuberculosis and Lung Disease, Kampala, Uganda; ^2^International Union Against Tuberculosis and Lung Disease, Paris, France; ^3^National Tuberculosis and Leprosy Program, Ministry of Health, Kampala, Uganda; ^4^Division of Global HIV and Tuberculosis, CDC Uganda; ^5^Division of Global HIV and Tuberculosis, Global Health Center, CDC.

**Summary**

**What is already known about this topic?**

Household contacts of persons with pulmonary tuberculosis (TB) are at increased risk for infection and disease. Effective interventions to improve TB case finding and to increase use of preventive treatment are lacking.

**What is added by this report?**

This pilot project enrolled 521 index patients with TB disease at six health facilities in Uganda. Among 1,913 household contacts, 90.9% were screened for TB; 1,239 initiated preventive treatment, approximately 95% of whom completed it. Eighty new cases of TB were diagnosed. The approach included home visits, chest radiography, adherence counseling, and travel reimbursements.

**What are the implications for public health practice?**

Global scale-up of this household contact approach might help reach global TB elimination goals.

## Abstract

To help achieve the End TB Strategy target of a 90% reduction in tuberculosis (TB) incidence by 2030, member states of the United Nations High-Level Meetings on TB called for improving provision of TB preventive treatment (TPT) for household contacts of persons with TB, who are at increased risk for infection and disease. However, TPT use among household contacts worldwide remained at 21% in 2023. The International Union Against Tuberculosis and Lung Disease, the Uganda Ministry of Health, and CDC piloted a comprehensive approach for increasing case finding and TPT coverage among household contacts of persons with TB. During November 1, 2023–September 30, 2024, a total of 521 index patients with TB disease were registered at six health facilities in Uganda. Home visits to index patients identified 1,913 household contacts, 1,739 (91.0%) of whom underwent TB symptom screening at home; 321 (18.5%) reported TB symptoms. Of 309 (96.3%) persons with TB symptoms who were further evaluated, 284 (91.9%) provided a sputum specimen for laboratory testing, including 270 (84.1% of those with symptoms) who did so during the home visit; 214 (69.3%) underwent chest radiography. Overall, 80 TB cases were diagnosed; in 61 (76.3%) persons, the diagnosis was based on radiographic findings. Among 1,496 HHCs eligible for TPT, 1,239 (82.8%) initiated treatment and 1,178 (95.1%) completed it. Global scale-up of this approach might help reach global TB elimination goals.

## Introduction

In 2023, an estimated 10.8 million persons fell ill with tuberculosis (TB), and 1.25 million died from the disease worldwide (*1*). TB risk is increased in household contacts (HHCs) of persons with TB; screening HHCs for TB and providing TB preventive treatment (TPT) are important approaches for achieving the End TB Strategy’s target of a 90% reduction in incidence by 2030 (*2*). At the United Nations High-Level Meetings on TB in 2018 and 2023, member states called for improving TPT coverage and committed to providing TPT to 30 million HHCs by 2027. However, in 2023, TPT use among HHCs remained at 21% (*1*). Previous studies (*3*–*6*) have documented suboptimal TB case finding and TPT use among HHCs due to implementation challenges including lack of home visits, indirect symptom screening via index patients, and limited ability to motivate HHCs with presumptive TB to visit health facilities for further evaluation. Programmatic solutions are urgently needed to overcome these challenges.

Uganda is a high TB–incidence country, with 198 cases per 100,000 population each year (*1*). Although national guidelines recommend evaluating all HHCs of persons with pulmonary TB and initiating TPT in those without disease or contraindications regardless of age (*7*), implementation gaps remain (*8*,*9*). This report describes the outcomes of an intervention to increase TB case finding and TPT use among HHCs of persons with pulmonary TB in Uganda.

## Methods

### Project Setting and Population

This pilot project was implemented in six high-volume TB clinics in four public and two private, not-for-profit health facilities in Uganda (Supplementary Table 1, https://stacks.cdc.gov/view/cdc/176833#tabs-3), where chest radiography (CXR) could be performed on-site or through referral to a nearby facility. An index TB patient was defined as a person of any age with new or recurrent bacteriologically confirmed pulmonary TB (*7*). An HHC was defined as a person who shared the same enclosed living space with the index patient for ≥1 night, or for frequent or prolonged daytime periods, during the 3 months before the start of current treatment (*10*). All index patients recorded in the selected facilities’ TB register during November 1, 2023–September 30, 2024, and their respective HHCs were included.

### Case Finding and Management of HHCs with TB Disease

**FIGURE 1:**
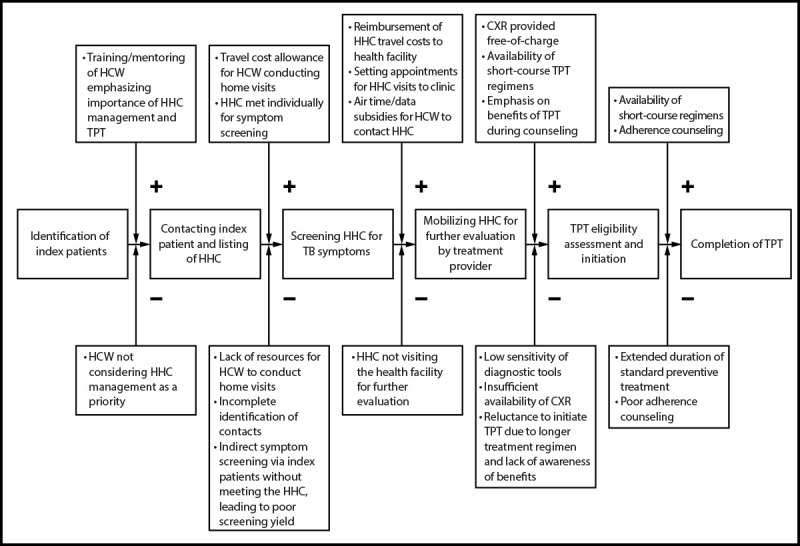
Interventions designed to mitigate challenges in the management of household contacts of persons with pulmonary tuberculosis — six health facilities, Uganda, 2023–2024 **Abbreviations:** CXR = chest radiography; HCW = health care worker; HHC = household contact; TB = tuberculosis; TPT = TB preventive treatment.

HHCs were managed according to national guidelines (*7*). However, innovations were introduced to overcome the challenges highlighted in previous studies (Figure 1). Training of health care workers^†^ emphasized systematic screening of HHCs for TB disease using symptom assessments and CXR (interpreted by computer-assisted diagnosis software or a radiologist). Health care workers received travel and airtime subsidies to conduct home visits and contact HHCs. Home visits included symptom screening, sputum specimen collection for laboratory confirmation^§^ among symptomatic HHCs, and HIV testing (after [parental] consent) with rapid diagnostic tests. All HHCs, regardless of symptoms, were referred to facilities for a free-of-charge CXR; their travel costs were reimbursed.

### Management of HHCs Without TB Disease

HHCs without TB disease and without contraindications^¶^ to preventive treatment were offered TPT. To increase use and adherence, a short-course regimen (1 month of daily isoniazid and rifapentine) was made available, in addition to the 3 months of weekly isoniazid and rifapentine, and 6 months of daily isoniazid options, and intensive counseling sessions on the benefits of TPT were provided. On-site supervision and mentorship on guidelines and project implementation were provided quarterly to health care workers by the International Union Against Tuberculosis and Lung Disease staff members and District TB and Leprosy Supervisors.

### Data Sources

Structured data collection forms were used to extract project-specific and routinely collected demographic and clinical data from facility records and registers. Follow-up data was included until November 10, 2024.

### Data Analysis

Data were analyzed using Stata (version 16.0; StataCorp). Numbers and proportions for each stage of the TB care and TPT cascades were calculated. Means with SDs and medians with IQRs were used to summarize patient demographics and the duration (in days) for completing the various stages.

### Ethics

The Uganda Joint Clinical Research Centre’s Research Ethics Committee and National Council for Science and Technology granted ethics approval. This activity was reviewed by CDC, deemed not research, and conducted consistent with applicable federal law and CDC policy.**

## Results

**FIGURE 2:**
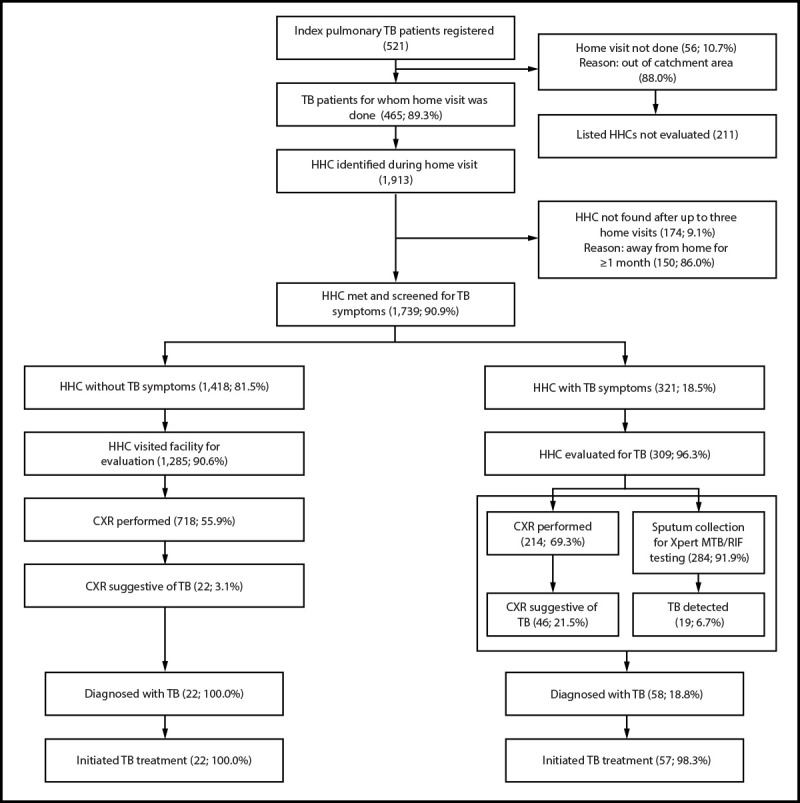
Tuberculosis diagnosis cascade among household contacts of persons with bacteriologically confirmed pulmonary tuberculosis*^,^^†^^,^^§^ initiated on treatment — six health facilities, Uganda, 2023–2024 **Abbreviations:** CXR = chest radiography; HHC = household contact; TB = tuberculosis; TPT = TB preventive treatment. * A bacteriologically confirmed TB case is one in which a biological specimen is positive by smear microscopy, culture, or molecular World Health Organization–approved rapid diagnostics such as Xpert MTB/RIF. ^†^ Evaluation for TB disease included symptom screening, a physical examination, CXR, and sputum specimen collection for bacteriologic testing; in asymptomatic patients, collection of a sputum specimen might not be possible. ^§^ Xpert MTB/RIF results were negative for 20 of 22 asymptomatic HHCs with TB. The clinician made a diagnosis of clinical TB and decided to give the patient a full course of TB treatment on the basis of CXR abnormalities.

Of 521 index patients registered in the six selected facilities November 1, 2023–September 30, 2024, 337 (64.7%) were men, 222 (42.6%) were living with HIV, and mean age was 34.6 years (SD = 11.7). Home visits were not possible for 56 (10.7%) index patients who listed 211 HHCs, primarily because they lived outside the facilities’ catchment areas. Among 465 (89.3%) index patients visited at home, 1,913 HHCs were identified, including 120 (6.3%) not previously listed by the index patient (Figure 2).

### TB Case Finding Among HHCs

Of 1,913 HHCs, 1,739 (90.9%) underwent TB symptom screening at home, 1,490 (85.7%) of whom were aged ≥5 years, and 900 (51.8%) of whom were men (Supplementary Table 2, https://stacks.cdc.gov/view/cdc/176833#tabs-3). During home visits, 1,391 HHCs (80.0%) consented to HIV testing; 48 (3.5%) received a positive test result. Among 472 HHCs of HIV-positive index patients, 37 (7.8%) received a positive test result for the virus.

Of the 1,739 screened HHCs, 321 (18.5%) reported TB symptoms^††^, 309 of whom (96.3%) underwent further evaluation; 284 provided a sputum specimen for laboratory confirmation, including 270 (84.1% of symptomatic HHCs) who did so during the home visit, and 214 (69.3%) underwent CXR. Of the 309, 58 (18.8%) received a diagnosis of TB disease, 19 of 58 (32.8%) were bacteriologically confirmed and 39 of 58 (67.2%) were clinically diagnosed.^§§^ Fifty-seven (98.3%) initiated TB treatment.

Among 1,418 (81.5%) asymptomatic HHCs, 1,285 (90.6%) visited a health facility for further evaluation; 718 (55.9%) underwent CXR. Of those, 22 (3.1%) had radiologic findings suggestive of TB; 20 received a clinical diagnosis of TB, and two were bacteriologically confirmed. All 22 initiated TB treatment.

Overall, 80 HHCs received a diagnosis of TB disease; CXR contributed to the diagnosis of 61 TB cases. A total of 21 TB cases (two in asymptomatic and 19 in symptomatic HHCs) were bacteriologically confirmed.

### Management of HHCs Without TB Disease

**FIGURE 3:**
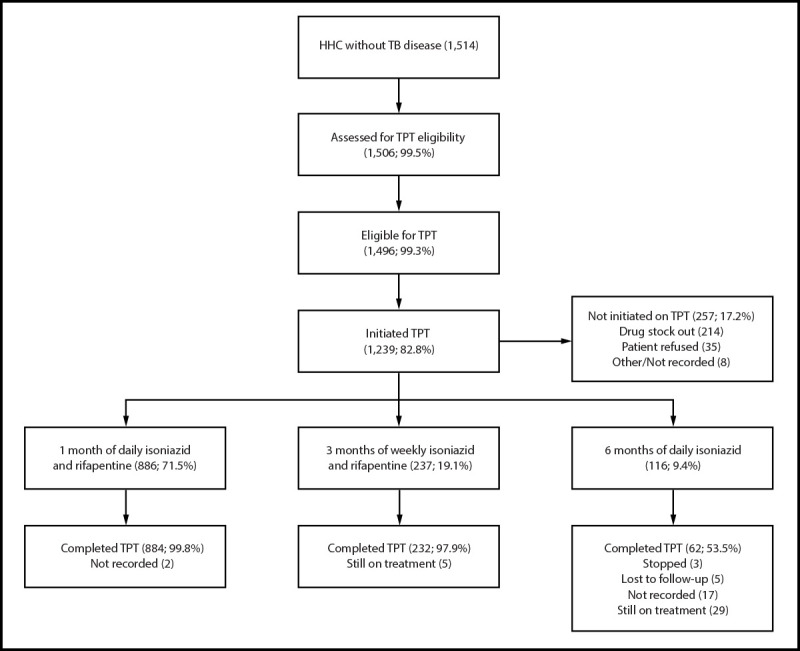
Tuberculosis preventive treatment cascade among household contacts* of persons with bacteriologically confirmed^†^ pulmonary tuberculosis initiated on treatment — six health facilities, Uganda, 2023–2024 **Abbreviations:** HHC = household contact; TB = tuberculosis; TPT = TB preventive treatment. * Includes 1,263 HHCs who reported no TB symptoms and 251 who reported symptoms but in whom TB was ruled out. ^†^ A bacteriologically confirmed TB case is one in which a biological specimen is positive by smear microscopy, culture, or molecular World Health Organization–approved rapid diagnostics such as Xpert MTB/RIF.

Of 1,514 HHCs who visited health facilities and did not receive a diagnosis of TB disease (Figure 3), 1,506 (99.5%) were assessed for TPT eligibility; 1,496 (99.3%) were eligible, and 1,239 (82.8%) initiated self-administered TPT, including 1,123 (91%) who received a short-course regimen. The majority of those who initiated TPT (78.3%) were aged ≥5 years. Of the 257 (17.2%) eligible HHCs who did not initiate TPT, 214 (83.3%) were unable because TPT drugs were out of stock at the health facilities; 35 (13.6%) declined TPT. Overall, 1,178 of 1,239 (95.1%) completed TPT. No severe adverse reactions to TPT drugs were reported.

Median duration from index patient registration to home visit was 1 (IQR = 0–8) day, and 2 days (IQR = 0–12 days) from registration to initiation of TB treatment or TPT (Supplementary Table 3, https://stacks.cdc.gov/view/cdc/176833#tabs-3). Among HHCs initiating TB treatment or TPT, 80.0% did so within 15 days of index patient registration.

## Discussion

This pilot project found that providing adequate resources to conduct home visits coupled with spot sputum collection, CXR, adherence counseling, and reimbursements of health care workers for travel and communication resulted in approximately 90% of HHCs of index TB patients being visited and screened for TB. Approximately 90% of symptomatic HHCs were promptly evaluated and 85% had sputum specimens collected during home visits.

Although efforts to provide access to CXR for asymptomatic HHCs yielded only 50.6% coverage, 22 (3.1% of those who underwent CXR) received a diagnosis of TB. Approximately three fourths of HHCs who received a TB diagnosis would have been missed had CXR not been performed. Screening tools that do not rely on eliciting symptoms, such as CXR, could prove essential to improve global efforts to diagnose asymptomatic TB and expand TPT among HHCs.

Among eligible HHCs, approximately 80% initiated TPT, in contrast with previous studies showing low TPT use (*5*,*6*). Most HHCs who initiated TPT were aged ≥5 years; this observation is encouraging because most programs have traditionally focused interventions for TPT in HHCs aged <5 years (*1*). Approximately 95% of HHCs who initiated TPT completed it, likely due to intensive counseling from health care workers emphasizing the benefits of TPT, availability of shorter TPT regimens, and adherence support during treatment.

### Limitations

This report is subject to at least two limitations. First, no control sites were included for comparison. Second, TB infection testing was unavailable to assess TPT eligibility. Thus, results reflect a programmatic approach in which HHCs without TB disease were assessed for TPT eligibility regardless of infection status, which might have led to overtreatment.

### Implications for Public Health Practice

This project demonstrated an effective intervention for closing gaps in HHC management that could help reach global TB elimination goals. The use of CXR is critical in diagnosing HHCs with TB disease and identifying those eligible for TPT. 

## Footnotes

* These authors contributed equally to this report.

† TB nurses and community health workers.

§ A bacteriologically confirmed TB case is one in which a biological specimen is positive by smear microscopy, culture, or molecular World Health Organization–approved rapid diagnostics such as Xpert MTB/RIF.

¶ Per national guidelines, contraindications include a history of multidrug-resistant TB treatment; acute or chronic liver disease; alcohol dependence; known or suspected hypersensitivity to isoniazid, rifamycin, or other TPT medicines; a history of convulsions or psychosis; moderate or severe peripheral neuropathy; and concomitant medications: anticonvulsants, antifungals, selective serotonin re-uptake inhibitor antidepressants, anticoagulants, and others.

** 45 C.F.R. part 46, 21 C.F.R. part 56; 42 U.S.C. Sect. 241(d); 5 U.S.C. Sect. 552a; 44 U.S.C. Sect. 3501 et seq.

†† Symptoms included current cough, hemoptysis, fever, night sweats, and weight loss (or poor weight gain in children aged <5 years).

§§ A clinically diagnosed TB case is an illness in a patient who does not fulfill the criteria for bacteriological confirmation but for which the patient has received a diagnosis of active TB by a clinician or other medical practitioner who has decided, based on CXR abnormalities and other clinical considerations, to give the patient a full course of TB treatment.
